# The Unknown Process Osseointegration

**DOI:** 10.3390/biology9070168

**Published:** 2020-07-16

**Authors:** Nansi López-Valverde, Javier Flores-Fraile, Antonio López-Valverde

**Affiliations:** Department of Surgery, University of Salamanca, Instituto de Investigación Biomédica de Salamanca (IBSAL), 37007 Salamanca, Spain; nlovalher@usal.es (N.L.-V.); j.flores@usal.es (J.F.-F.)

**Keywords:** osseointegration, foreign-body reaction, bone remodeling, immuno/neuromodulatory techniques

## Abstract

Although it was already described more than fifty years ago, there is yet no in-depth knowledge regarding the process of osseointegration as far as its mechanism of action is concerned. It could be one of the body’s ways of reacting to a foreign body, where the individual’s immune response capacity is involved. It is known that the nervous system has an impact on bone health and that the role of the autonomic nervous system in bone remodeling is an attractive field for current research. In the future, immuno/neuromodulatory techniques will open new and exciting lines of research.

Although the process of osseointegration was first described by Brånemark and colleagues [1], 50 years later, the real mechanism of this process, remains unknown and has not been studied in depth. The model proposed by Koka and Zarb marked genetics as one of the patient’s inherent variables, necessary, to achieve “sufficient” and lasting results [2].

Among the proposed theories, two have acquired particular interest: “*foreign-body reaction*”, which interprets osseointegration from the point of view of adverse immune processes [3]; and the so-called by certain authors “*brain-bone axis*” theory [4].

## 1. Foreign-Body Reaction

Contact between any foreign body and vital tissue can trigger the activation of the immune/inflammatory systems, activating defense cells: neutrophils, lymphocytes, monocytes and proinflammatory macrophages, among others. Osteoclasts have a variety of specific functions in bone tissue, many of them unknown. Their function, regarded, exclusively, as bone remodeling, has been reassessed in the last decade, recognizing them as cells that are capable of secreting cytokines that share a common origin with others of the immune system. Kiesel and colleagues [5] proposed a very attractive hypothesis according to which osteoclasts would be antigen-presenting cells in the bone marrow, participating in the recruitment and maintenance of CD8+ T lymphocytes.

However, in addition to osteoclasts, cells derived from the monocyte/macrophage lineage, are one of the first cell types that come into contact with biomaterials implanted in bone tissue, alongside osteoclasts, and certain foreign body giant cells (multinucleated giant cells, MNGCs) take part in the encapsulation or rejection of the implant material [6]. In vitro research has proven that macrophages can differentiate into osteoclasts or into MNGCs [7]; however, the role of both cell types, osteoclasts and MNGCs, remains unknown to researchers, even though there is growing evidence that MNGCs can play a specific role in the integration of biomaterials with the host bone [8]. On the other hand, neither is there a clear answer to whether MNGCs would remain active throughout the entire life of the foreign body or whether their activity would disappear over time, in which case bone tissue repair would evolve into a condition of chronic inflammation, that would end with the consequent destruction of the tissue [6,9].

## 2. Brain-Bone Axis

Recent studies, have shown that the nervous system plays an essential role in bone regeneration and remodeling [10,11]. The participation of the autonomic nervous system, especially, the sympathetic nervous system (SNS), in the modulation of bone remodeling, is awakening growing interest among researchers. An intact autonomic nervous system contributes to the maintenance of healthy bone tissue, whereas its alteration could induce abnormal bone remodeling and its association with clinical conditions such as postmenopausal osteoporosis, adolescent idiopathic scoliosis, complex regional pain syndrome or depression-induced osteoporosis [12,13]. Bone remodeling would be controlled by the hypothalamus through a process involving adrenergic nerves and neurotransmitters [14]. The growth hormones secreted by the pituitary gland, controlled by the hypothalamus, could induce osteoblast and osteoclast proliferation, playing an essential role in the bone formation-destruction balance [15].

The discovery of glucose-sensing neurons in the arcuate nucleus of the hypothalamus would be compatible with the possibility of the brain monitoring the skeleton’s condition [16].

The skeleton, is considered an active organ that sends information to others such as the brain. Lee and colleagues [17] proved that osteocalcin (the second most widely expressed protein in bone after collagen type I), behaves as a hormone, stimulating the release of insulin from the pancreatic *β*-cells. Other authors have reported that osteocalcin would regulate the brain, being crucial to the development of the hippocampus and cognitive functions [18]. On the other hand, neurotransmitters such as noradrenaline, serotonin and dopamine could take part in bone remodeling mechanisms [11,19]. Long-term use of central nervous system (CNS) depressants is known to cause decreased bone mass, resulting in osteoporosis and a higher risk for fractures. Gupta and colleagues [20] reported higher rates of implant failure in patients under antidepressant therapies, although they do not explain whether this is due to the drug or to the patients’ mental health itself, or perhaps even to a disruption of the CNS-bone remodeling axis.

While these considerations suggest that the skeleton’s homeostasis is regulated by the brain, it is necessary to conduct extensive research on the immune/inflammatory system, bone physiology and the brain’s role in bone growth and remodeling. All of this would contribute to the understanding and monitoring of the complex phenomenon of osseointegration.
